# Toscana Virus RNA in Sergentomyia minuta Flies

**DOI:** 10.3201/eid1208.060345

**Published:** 2006-08

**Authors:** Rémi N. Charrel, Arezki Izri, Sarah Temmam, Xavier de Lamballerie, Philippe Parola

**Affiliations:** * Université de la Méditerranée, Marseille, France;; † Faculté de Médicine Bobigny, Paris, France

**Keywords:** Toscana virus, sandflies, France, letter

To the Editor: Toscana virus (TOSV) (family Bunyaviridae, genus Phlebovirus) is an arthropodborne virus transmitted by sandflies. Reports of infections in travelers, clinical research, and epidemiologic studies have shown that TOSV affects the central nervous system and is a major cause of meningitis and encephalitis in Mediterranean countries in which the virus circulates (*1*). In central Italy, this virus is the primary cause of meningitis from May to October, far exceeding enteroviruses as a cause of disease (*2*). In other northern Mediterranean countries, TOSV is among the 3 most prevalent viruses associated with meningitis during the warm seasons (*1*). TOSV has recently been associated with human disease in France (*3**,**4*) and was originally isolated in Italy from Phlebotomus perniciosus, then from P. perfiliewi, but never from P. papatasi. TOSV has also been isolated from the brain of a bat in areas where P. perniciosus and P. perfiliewi were present, but no hemagglutination-inhibiting antibodies were found in sera from these bats (*5*). In Spain, 2 isolates of TOSV were recovered from 103 pools of sandflies; sequence analyses showed that they were genetically divergent from the Italian strains (*6*). To date, TOSV had not been isolated from sandflies collected in France.

In July 2005, a total of 123 Sergentomyia minuta were collected in a 4-day period near Marseille, southeastern France. This work was part of a larger collaborative study, the results of which will be published separately. CDC miniature light traps (John W. Hock Co., Gainesville, FL, USA) were adapted to sandflies with an ultra-fine mesh. Traps were hung 1–2 m above the ground. They were placed in the late afternoon inside or near animal housing facilities (for chickens, rabbits, goats, or horses) in the suburbs of Marseille for 4 successive nights. In these areas, large numbers of geckos were noticed. Each morning, sandflies were collected, identified morphologically, and placed in 1.5-mL Eppendorf tubes. S. minuta flies were identified by appearance, and genus was confirmed by sequence analysis, as previously reported (*7*).

Five pools of the captured S. minuta were prepared with a maximum of 30 flies per pool. They were ground in 20% fetal bovine serum–enriched phosphate-buffered saline in a Mixer Mill MM300 (Qiagen, Courtaboeuf, France) with one 3-mm tungsten bead and clarified by low-speed centrifugation. We used 200 μL supernatant for total RNA purification onto the MagNAPure platform with the MagNA Pure LC RNA High Performance Kit (Roche Diagnostics, Meylan, France). We used 10 μL RNA suspension for reverse transcription PCR, with primers targeting either a consensus sequence for the phlebovirus polymerase gene (L RNA segment) or Toscana virus (*8*) and the nucleoprotein (N) gene (S RNA segment) specifically (*9*).

Only 1 TOSV-positive pool was observed with primers specific to TOSV polymerase and N genes, respectively. A positive result was observed with primers NPhlebo2+ and ATos2, previously found to target polymerase genes of a range of phleboviruses (*8*). This result was confirmed by sequence analysis (GenBank accession no. DQ195277), which showed 82.8% and 96% identity at the nucleotide and amino acid level, respectively, with a TOSV isolate from Italy (GenBank accession no. X68414). The same pool also tested positive with primers (5´-CGTRGCAGCCACYTCATTAG-3´ and 5´-GTGTCGGCYGCSTTTGTTCC-3´) designed in this study from the alignment of the 13 sequences of TOSV retrieved from GenBank (accession nos. are shown in the Figure). Comparing the sequence of this 272-bp PCR product with homologous sequences of selected phleboviruses available in the GenBank database showed 97.4%, 87.1%–88.2%, and 78.7% identity at the nucleotide level with TOSV strains isolated in Italy, TOSV isolated in Spain, and sandfly fever Naples virus (Sabin strain), respectively. Phylogenetic analyses of the N gene indicated that this virus clustered with TOSV strains circulating in Italy and Spain but is most closely related to isolates from Italy (Figure). Comparative analysis within the polymerase gene confirmed these data, but distance analysis with sequences of Spanish TOSV was not possible because genetic data were lacking in public databases. The remaining 400-μL volume of sandfly material was used to attempt virus isolation in Vero cells and by intracerebral injection of 2-day-old suckling mice, but no virus was recovered.

**Figure:**
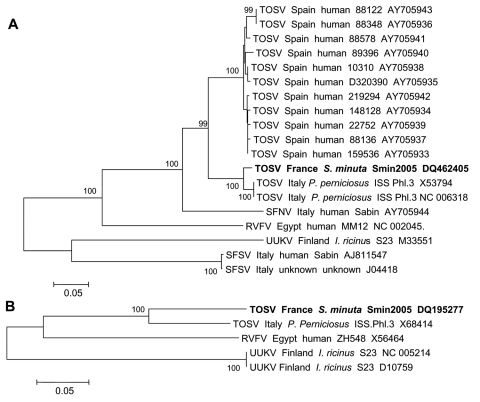
Phylogenetic analysis of Toscana virus (TOSV) from *Sergentomyia minuta* based on 272- and 128-nucleotide sequences in the nucleoprotein (A) and polymerase (B) genes of viral RNA, respectively. Distances and groupings were determined by the p-distance algorithm and neighbor-joining method with the MEGA software program. Bootstrap values >95% are indicated and correspond to 500 replications. For each sequence, the following information is indicated: virus/geography/host/isolate name/GenBank accession no. SFSV, sandfly fever Sicilian virus; SFNV, sandfly fever Naples virus; RVFV, Rift Valley fever virus; UUKV, Uukuniemi virus. Sequences determined in this study are shown in **boldface**.

To our knowledge, this is the first time that TOSV has been detected in phlebotomine flies other than P. perniciosus and P. perfiliewi. S. minuta was identified with morphologic keys and confirmed by sequencing a portion of the 28S gene (*7*). Sergentomyia spp. have been reported to be infected by a variety of different RNA viruses, such as Chandipura (*10*), Saboya (*11*), Tete, and 2 unclassified viruses (ArD 95737 and ArD 111740). However, S. minuta feed on reptiles but not on humans, which may prevent them from being vectors of human infection. Additional studies are needed to better understand the role of Sergentomyia spp. and other arthropods in the ecology of TOSV. Whether TOSV also circulates in Phlebotomus spp. in France remains to be determined, but the evidence for human infections with this virus shows that more extensive investigations are needed to understand the role of this arbovirus in neurologic diseases in the Mediterranean.
